# Elements of healthcare delivery required to facilitate the clinical governance of hospital pharmacy services: a document review

**DOI:** 10.1186/s12961-025-01378-w

**Published:** 2025-08-04

**Authors:** Huri Balikubiri, Anna Kemp-Casey, Andre Q. Andrade, Elizabeth E. Roughead

**Affiliations:** 1 Quality Use of Medicines and Pharmacy Research Centre, Clinical and Health Sciences, University of South Australia, Adelaide, SA Australia; 2 SA Pharmacy, Adelaide, SA Australia

**Keywords:** Clinical governance, Hospital pharmacy services, Healthcare quality

## Abstract

**Background:**

Clinical governance of hospital pharmacy services aims to ensure the delivery of pharmaceutical care that maximizes positive outcomes for patients. Good clinical governance incorporates service-related data to evaluate and improve care delivery. National-level clinical governance frameworks available in Australia provide limited information on auditable metrics to evaluate hospital pharmacy services. This study reviews healthcare governance documents to determine the goals and desirable elements of healthcare delivery and the information to measure to inform the clinical governance of hospital pharmacy services.

**Methods:**

A purposive online search was conducted to identify healthcare governance documents describing the clinical governance approach for general healthcare and hospital pharmacy services in Australia, New Zealand, England, Canada and the United States. Documents for general healthcare settings were reviewed to identify the goals and desirable elements of healthcare delivery. Pharmacy governance documents were then reviewed to contextualize the identified elements of care to hospital pharmacy services and to determine the information to measure to inform clinical governance.

**Results:**

The objective of delivering quality healthcare was included in all documents. Quality healthcare was described in the documents as healthcare that is patient-centred, safe, effective, integrated, timely, equitable, efficient and reliable. These factors were considered desirable elements of healthcare delivery that promote positive patient outcomes. The performance measures to evaluate and improve hospital pharmacy services delivery covered pharmacists’ clinical activities, pharmaceutical care outcomes, stakeholder feedback and medication incidents and risks.

**Conclusions:**

Measuring the extent to which the desirable elements of care are evident in the performance of clinical pharmacists’ activities and the associated outcomes of care may provide useful information to facilitate the clinical governance of hospital pharmacy services.

**Supplementary Information:**

The online version contains supplementary material available at 10.1186/s12961-025-01378-w.

## Background

Governance refers to the systems by which corporations are directed and controlled [1]. For healthcare organizations, clinical governance is an integrated component of their corporate governance that is concerned with the delivery of healthcare to maximize positive outcomes for patients [2–4]. According to the Australian Commission on Safety and Quality in Health Care (ACSQHC), clinical governance encompasses the leadership behaviours, policies, procedures, monitoring and quality improvement systems implemented in healthcare organizations to evaluate and improve care delivery [3].

It is widely recognized that the delivery of good healthcare relies on the integration of effective leadership and learning systems into the clinical governance structure implemented in healthcare organizations [2–8]. Through leadership, organizational goals and the roles and responsibilities of members are strategically established and communicated [3]. Continuous monitoring of care delivery through learning systems is recommended to enable regular evaluation and improvement of services [2]. Learning systems are implemented to promote organizational accountability by monitoring processes involved in the delivery of care and indicating the extent to which organizational goals are achieved [6]. Learning systems can identify strengths and weaknesses in healthcare delivery, providing useful information to guide service improvement initiatives [6].

In healthcare organizations, medications are the most utilized intervention [9]. Processes affecting medication use must be monitored to ensure the responsible use of medications and to promote positive patient outcomes [9]. In hospitals, the steps involved in the medication management pathway include the decision to prescribe a medication, the act of prescribing, reviewing the medication order, issuing the medication (dispensing, manufacturing and procuring), providing medication information, distributing and storing the medication, administering the medication, monitoring the use of the medication and communicating medication management information to enable the continuity of care [10]. These steps possess unique risks that may compromise the responsible use of medications in hospitals [11].

Hospital pharmacies are implemented to reduce the risks presented at various stages of the medication management pathway. Hospital pharmacies offer many services that aim to optimize patient outcomes by supporting the responsible use of medicines throughout the patient care process [10, 12, 13]. These services include clinical, distribution, production, education, research, administration and quality improvement functions [14]. Clinical pharmacy services include activities such as obtaining a medication history or providing discharge medication counselling. These services are provided to patients by pharmacy staff and influence the management of a patient’s medications and overall pharmaceutical care [10]. Hospital pharmacy administration and quality improvement services oversee clinical governance processes by coordinating and directing the delivery of clinical services [1, 2, 14].

National or locally developed frameworks may be utilized by hospital pharmacies to guide the implementation of clinical governance. These frameworks recommend organizational goals, systems, processes, roles and responsibilities for healthcare organizations to implement to deliver healthcare that promotes positive patient outcomes [15, 16]. Previous research investigating the implementation of clinical governance in clinical practice has highlighted the importance of effectively utilizing data that reflect key organizational processes to enable meaningful evaluation of services and the achievement of their specified clinical care goals [16–20]. However, in Australia, clinical governance frameworks that outline auditable measures to evaluate hospital pharmacy services are scarce. The existing clinical governance framework for pharmacy services in Australia is not specific to the hospital pharmacy setting and lacks recommendations for practice-related data specific to measure and monitor for effective service evaluation [2]. This absence of detailed guidance can lead to uncertainty among organizations about which information to incorporate into their learning systems to enable effective evaluation of clinical services and the achievement of clinical care goals [17, 19].

This study reviewed local and international clinical governance frameworks to identify the recommended goals and desirable elements of healthcare delivery. These factors were then contextualized to hospital pharmacy services to determine the practice-specific information recommended for measurement to inform clinical governance on the attainment of organizational objectives for clinical care delivery.

## Methods

### Design

A qualitative review of healthcare governance documents was performed. Documents from healthcare organizations in Australia, Canada, England, New Zealand and the United States were purposely sought. These countries were included because they were highlighted in the Pharmaceutical Society of Australia’s Clinical Governance Principles for Pharmacy Services (2018) as international examples of jurisdictions that have implemented leading clinical governance frameworks in hospital and primary care settings [2]. These countries also have a similar economic development level to Australia (high income) and have documents written in English [21].

### Document selection

Documents were collected through two structured rounds of online searching. The first round focused on identifying national clinical governance frameworks relevant to general healthcare settings by searching the websites of national healthcare organizations in the selected countries. The second round targeted hospital pharmacy-specific governance documents, such as governance frameworks and practice standards or guidelines, sourced from the websites of national and multinational pharmacy organizations operating within those same countries.

The selection of documents was guided by the Australian Aged Care Quality and Safety Commission, which defines clinical governance frameworks as documents that outline organizational goals, the systems and processes established to achieve those goals and the roles and responsibilities of stakeholders [15]. A purposive search strategy was used, supported by predefined terms such as “clinical governance framework” and “quality and safety strategy”. The search terms used and websites visited are summarized in Additional File 1: Supplementary Table 1.

Documents were included if published in English and dated from 2010 onwards. This threshold was selected to ensure the relevance of included documents to contemporary healthcare practice. All documents were screened and selected by the lead author on the basis of the stated inclusion criteria.

### Document analysis

Document analysis was performed in two stages using a combination of conventional and directed qualitative content analysis, as outlined by Hsieh and Shannon (2005) [22]. Each stage involved multiple readings to firstly familiarize the investigator with the structure of the documents and then to classify their content. In the first stage, a conventional approach was used to analyse national clinical governance frameworks to inductively identify and categorize the desirable elements of clinical care delivery described in the texts. In the second stage, a directed content analysis was undertaken using the categories developed in Stage One as a coding framework. This involved identifying instances where the care elements were referenced or described in hospital pharmacy documents and interpreting how they applied to hospital pharmacy services. Additional categories were created if the pharmacy documents introduced new or distinct goals of care. Throughout this stage, recommendations on what to measure to facilitate the clinical governance of hospital pharmacy services were also extracted, categorized and mapped to the corresponding goals of clinical care.

### Ethics

The documents utilized in this study were publicly accessible, and this work did not require ethics review.

## Results

### Characteristics of the retrieved documents

A total of 16 documents were obtained; 5 documents described clinical governance frameworks in general healthcare settings [3, 5–8], and 11 documents described the governance frameworks, practices, standards or clinical guidelines for hospital pharmacy services [2, 10, 12, 13, 23–29]. The healthcare organizations whose websites were searched and the documents retrieved are summarized in Tables 1 and 2.

**Table 1:** Clinical governance documents retrieved from national healthcare organizations

Country	Healthcare organizations searched	Documents retrieved	Year
Australia	Australian Commission on Safety and Quality in Health Care (ACSQHC)	*National Model of Clinical Governance Framework* (ACSQHC)	2017
New Zealand	New Zealand Health Quality and Safety Commission (NZHQSC)	*Clinical Governance – Guidance for Health and Disability Providers* (NZHQSC)	2017
England	Health Education England Department of Health and Social Care National Quality Board (NQB) National Health Service Care Quality Commission National Institute for Health and Care Excellence (NICE) The Health Foundation (HF)	*A Shared Commitment to Quality for Those Working in Health and Care Systems* (NQB)	2021
Canada	Health Standards Organization (HSO) Canadian Patient Safety Institute (CPSI) Health Canada Healthcare Excellence Canada	*The Canadian Quality & Patient Safety Framework for Health Services* (HSO and CPSI)	2020
United States	Institute for Healthcare Improvement (IHI) Safe and Reliable Healthcare Agency for Health Research Quality The Centres for Medicare & Medicaid Services The Joint Commission (JC)	*A Framework for Safe, Reliable, and Effective Care* (IHI)	2017

**Table 2:** Hospital pharmacy documents retrieved from national and multinational pharmacy organizations

Country	Pharmacy organizations searched	Documents retrieved	Year
Australia	Pharmaceutical Society of Australia (PSA) Society of Hospital Pharmacists of Australia (SHPA)	*Clinical Governance Principles for Pharmacy Services* (PSA) *Standards of Practice for Clinical Pharmacy Services* (SHPA)	2018 2013
New Zealand	Pharmaceutical Society of New Zealand (PSNZ) New Zealand Hospital Pharmacy Association (NZHPA) Pharmacy Council of New Zealand Pharmacy Guild of New Zealand	*National Pharmacist Services Framework* (PSNZ) *Standards of Practice for New Zealand Hospital Clinical Pharmacy Services* (NZHPA)	2014 2019
England	Royal Pharmaceutical Society (RPS) General Pharmaceutical Council (GPC)	*Professional Standards for Hospital Pharmacy Services* (RPS)	2022
Canada	Canadian Society of Hospital Pharmacists (CSHP) Canadian Council on Continuing Education in Pharmacy (CCCEP) Canadian Pharmacy Association (CPhA) National Association of Pharmacy Regulatory Authorities (NAPRA)	*Pharmacy Practice in Hospitals and Other Collaborative Healthcare Settings* (CSHP) *Hospital Pharmacists: Information Paper on Enhancing Quality and Safety in Medication Use* (CSHP)	2016 2010
United States	American Society of Health Systems Pharmacists (ASHP) American College of Clinical Pharmacists (ACCP) American Pharmacists Association	*ASHP Guidelines: Minimum Standards for Pharmacies in Hospitals* *Standards of Practice for Clinical Pharmacists* (ACCP)	2013 2014
Multinational	International Pharmaceutical Federation (FIP) The European Association of Hospital Pharmacists (EAHP)	*Revised FIP Basel Statements on the Future of Hospital Pharmacy* (FIP) *European Statements of Hospital Pharmacy* (EAHP)	2014 2014

### Elements of care identified in national clinical governance frameworks

The desirable elements of healthcare delivery identified from the national clinical governance frameworks are presented in Additional File 2: Supplementary Table 2. The frameworks stated a common goal of promoting the delivery of healthcare that is high quality, patient-centred, safe, effective and integrated [3, 5–8]. The frameworks also stated that healthcare delivery must be timely and equitable in access, reliable, and efficient [3, 5–8].

The term “quality” was widely used across the clinical governance frameworks as a parent term for healthcare delivery that incorporated many of the other desirable elements of care identified. Four frameworks defined healthcare quality, but their definitions were inconsistent, as presented in Table 3. All frameworks implied that quality included the elements of patient-centredness, safety and effectiveness [5–8]. Other elements of care, such as timeliness, equity, integration, efficiency and reliability, were mentioned to varying degrees. In this study, quality was thus categorized as the overarching goal of healthcare delivery, composed of several subcomponents. Table 4 presents paraphrased descriptions of the elements of care that constitute healthcare quality derived from the documents reviewed.

**Table 3:** Desirable elements of clinical care delivery described in national clinical governance frameworks as components of quality in healthcare

	*Clinical Governance – Guidance for Health and Disability Providers* [7]	*A Shared Commitment to Quality for Those Working in Health and Care Systems* [5]	*The Canadian Quality and Patient Safety Framework for Health Services* [8]	*A Framework for Safe, Reliable, and Effective Care* [6]
Patient-centred	X	X	X	X
Safe	X	X	X	X
Effective	X	X	X	X
Equitable	X	X	X	
Efficient	X	X		X
Integrated		X	X	
Reliable				X
Timely			X

**Table 4:** Paraphrased definitions of the desirable elements of healthcare delivery that constitute quality in healthcare delivery

Care elements	Definitions from national clinical governance frameworks	Descriptions from hospital pharmacy documents
Patient-centred	-Care is tailored to the patients’ needs and considers matters that are important to them -Patients and consumers are encouraged and empowered to participate in healthcare planning, design, measurement and evaluation -Care is delivered with humility in a holistic, dignified and respectful manner [3, 5–8]	-The choice of medication therapy accounts for the patient’s needs and preferences -Patients and consumers are encouraged and empowered to actively participate in decision-making about their medication therapy, and monitoring -Patients and consumers are involved in the planning and design of pharmacy services [2, 12, 13, 23, 29]
Safe	-Care is delivered in a manner that reduces the risk of misadventures and preventable harm -Risk is managed to maximize benefits and minimize harm for the recipients and providers [3, 5–8]	-The choice of medication therapy for patients is informed by what has been proven by scientific and/or clinical evidence to be least likely to cause harm [2, 12, 13, 23, 29]
Effective	-The care provided is guided by evidence that suggests it will facilitate health promotion and disease prevention -The delivery of care is guided by the best evidence-based practice unless patient-specific considerations are contraindications [3, 5–8]	-The choice of medication therapy for patients is informed by what has been proven by scientific and/or clinical evidence to be effective for the management of a particular ailment [12, 23]
Timely	-Care is delivered to patients when needed with minimal delay -Care is delivered to patients at the right time [6–8]	-Pharmacists should review medication orders as soon as possible -Patients and healthcare providers should have timely access to pharmacy services when needed [2, 10, 12, 13, 23, 27, 29]
Equitable	-Care can be accessed by all who need it irrespective of age, gender, race/ethnicity, geography and socioeconomic status [5–8]	-The patient’s health needs determine the extent to which pharmaceutical care is provided. The needs of people from all backgrounds should be equally addressed [29]
Integrated	-Care is delivered in a collaborative and multidisciplinary team setting to reduce the risk of task fixation -Care is collaboratively designed, delivered and evaluated through partnerships between healthcare providers to deliver the best outcomes for their patients [3, 5–8]	-A collaborative multidisciplinary approach is adopted which includes input from patients, consumers and other healthcare professionals in the management of medications [2, 10, 12, 13, 26, 27, 29]
Reliable	-Care is delivered in a way that is consistently safe, efficient and person-centred, and minimizes unwarranted variations [5–8]	-Pharmacy organizations should consistently provide clinical services to the standards described in their guidelines [23, 27, 29]
Efficient	-Care provides the best possible outcomes considering resource limitations [5–8]	-Resources are utilized optimally to achieve the desired outcomes [23]

Some frameworks expressed the need for healthcare to be delivered appropriately [3, 8]. However, the term “appropriate” was not prominent among the frameworks as its subcomponents (effective and patient-centred) were more frequently used. Consequently, the term “appropriate” was not included when categorizing the desirable elements of care, but instead, its subcomponents were included. Similarly, the terms “timely” and “equitable” were included in the categorization of desirable elements of care as their parent term (accessible) was not broadly used among the frameworks [7, 8].

### Elements of care identified in pharmacy governance documents

The desirable elements of healthcare delivery identified in hospital pharmacy documents were categorized similarly to those identified in national clinical governance frameworks (Additional File 2: Supplementary Table 3). No additional goals or desirable elements of care were identified in these documents to warrant alternative categorization. All pharmacy documents expressed the goals of delivering pharmaceutical care that is high quality, patient-centred, safe and effective [2, 10, 12, 13, 23–29]. To varying degrees, these documents also stated the need for care to be integrated, timely, equitable, reliable and efficient [2, 10, 12, 13, 23–29]. Delivering quality pharmaceutical care was also identified as the overarching goal in these documents, with elements of patient-centeredness, safety and effectiveness most frequently referenced as subcomponents of quality. Table 5 presents all the elements of care that were attributed to quality in these documents, and their paraphrased definitions are summarized in Table 4.

**Table 5:** Desirable elements of clinical delivery described in hospital pharmacy documents as components of quality in healthcare

	National Pharmacist Services Framework 2014 [25]	Standards of Practice for Clinical Pharmacy Services [10]	Pharmacy Practice in Hospitals and Other Collaborative Healthcare Settings [29]	ASHP Guidelines: Minimum Standards for Pharmacies in Hospitals [27]	Professional Standards for Hospital Pharmacy Services [28]	Clinical Governance Principles for Pharmacy Services 2018 [2]	Hospital Pharmacists: Information Paper on Enhancing Quality and Safety in Medication Use [23]
Patient-centred	X	X	X		X	X	X
Safe	X	X		X	X	X	X
Effective	X	X		X	X	X	X
Equitable						X	X
Efficient				X		X	X
Integrated							
Reliable			X			X	
Timely						X	X

### Measurements to inform the clinical governance of hospital pharmacy services

All documents evaluated highlighted the need for healthcare organizations to regularly measure and monitor the quality of care to support service evaluation and continuous improvement [2, 3, 5–8, 10, 12, 13, 23–25, 27–29]. Hospital pharmacy documents were reviewed to determine the practice-specific information recommended to measure. These documents emphasized the need to focus data capture on the clinically relevant aspects of hospital pharmacy services. The recommended information to measure was broadly categorized into (I) the provision of clinical services, (II) medication incidents and risks, (III) stakeholder feedback and (IV) outcomes of care (Table 6).

**Table 6:** Information to measure to inform clinical governance of hospital pharmacy services

Category	Information
Provision of clinical services	Clinical activities completed by pharmacy staff
	Individual clinician’s performance
	Adherence to clinical protocols and guidelines
Medication incidents and risk	Medication errors
	Adverse drug events
	Adverse drug reactions
	Medication-related incident reports
	Patient’s risk of medication misadventure
Stakeholder feedback	Consumer experience of care
	Staff feedback
Outcomes of care	Mortality
	Readmissions
	Medication-related readmissions
	Length of stay
	Cost of care

Measuring specific activities and information within the broader categories of recommended data capture was considered useful in demonstrating the value of hospital pharmacy services and facilitating quality improvement [2, 10, 12, 13, 23–25, 27–29]. This was emphasized for the measurement of clinical activities performed by pharmacy staff, as they were indicated to be associated with positive patient outcomes [10, 23–25]. The clinical activities referenced in these documents were also extracted and mapped to components of quality in clinical care delivery that they were associated with, as presented in Table 7. Most activities were linked to improvements in safety, while no associations were identified for enhancements in the reliability or equitability of care. The paraphrased definitions of these clinical activities are summarized in Additional File 3: Supplementary Table 4.

**Table 7:** Clinical pharmacy activities associated with components of quality care delivery

Classification	Activity	Patient-centred	Safe	Effective	Timely	Equitable	Efficient	Reliable	Integrated
Patient-specific pharmacist activities	Medication history		x [23]				x [23]		
	Medication reconciliation		x [24]	x [24]					
	Medication review		x [24]	x [24]					
	Clinical review		x [24]	x [24]					
	Adverse drug reaction management		x [23]						
	Medication optimization and management plans		x [24]	x [24]					
	Medication prescribing	x [24]	x [24]		x [24]				x [24]
	Medication administration								
	Therapeutic drug monitoring		x [23]						
	Documentation of care delivery								
	Patient counselling and education	x [24]							
	Provision of discharge services		x [24]	x [24]					
Patient-specific health team activities	Medical rounds		x [23, 24]		x [24]		x [23, 24]		x [23, 24]
	Multidisciplinary meetings								
Non-patient-specific pharmacist activities	Development of medication guidelines and protocols		x [23]				x [23]		
	Research		x [23]						
	Drug use evaluations		x [23]				x [23]		
	Antibiotic stewardship								
	Medication information to other health services		x [23]				x [23]		
	In-service training and education		x [23]				x [23]		

## Discussion

This review of healthcare governance documents provides insight into the recommended goals and desirable elements of clinical care delivery for hospital pharmacy services, as well as the information to measure to facilitate clinical governance. The national clinical governance frameworks supported the provision of quality healthcare, defined as patient-centred, safe, effective, integrated, timely, equitable, efficient or reliable. These elements were stated as care objectives or addressed as important components of clinical care delivery that promote positive patient outcomes. These findings were consistent with the review of pharmacy documents; however, in these documents, the elements of care were discussed in the context of governing clinical pharmacy service to promote the responsible use of medications in hospitals.

The alignment between national clinical governance frameworks and hospital pharmacy guidance documents is anticipated. National frameworks are usually developed to provide overarching clinical governance principles and directives for general healthcare settings [3, 18, 30]. Organizations within specific healthcare settings utilize these frameworks to develop practice standards and clinical guidelines to facilitate the implementation of clinical governance [2, 3, 10, 18]. These documents serve as extensions of broader national directives, contextualizing them to specific organizational environments. The alignment in goals suggests coherence among policy-makers across different levels of care, reflecting shared values and objectives for clinical care delivery.

The primary objective promoted across all the documents was the delivery of quality healthcare; however, the term “quality” was not consistently defined. This coincides with research investigating clinical governance in various healthcare settings, which highlights its consistent association with the quality of care [18–20, 31, 32]. Moreover, previous research exploring the definition of quality in healthcare indicates that the concept is broad and subjective and may not be well-defined within and across organizations [33–35]. Past research has also revealed that the desirable elements of care outlined in this study have been included in the past definitions of quality to varying degrees [33]. The observed differences in the interpretation of the term “quality” among the reviewed documents may stem from its inherently subjective nature, leading organizations to emphasize certain elements of care that align more closely with their specific values and priorities [35]. In the absence of a universal definition, quality in healthcare may be used to collectively describe the desirable elements of care identified in the documents reviewed.

Most of the evidence-based associations of clinical pharmacy activities and pharmaceutical care outcomes mentioned in the documents pertained to improvements in safety. There were relatively few associations with improvements in patient-centred, timely and integrated care, and none with reliability and equity. Similarly, many of the suggested measures were related to safety. Previous research investigating healthcare quality suggests that initiatives focusing solely on safety can inadvertently reduce the attention devoted to other elements of quality in healthcare assessment, which limits the ability to holistically evaluate quality [23, 36].

Moreover, substantial variability in access to clinical pharmacy services among patient populations has been reported, due to limited staffing or high workloads [37–41]. Consequently, the prioritization of patients who are most in need of pharmacy services is integral to pharmacy practice [39, 40, 42], which can be useful to monitor to understand the distribution of pharmacy services to vulnerable populations. Therefore, to promote a comprehensive assessment of healthcare quality, hospital pharmacy organizations could devote more attention to evaluating elements of care that may be less commonly investigated [23].

Few documents attributed individual clinical pharmacy activities to specific health outcomes or clinical care goals [23, 24]. Most documents broadly mentioned that clinical pharmacy activities provide evidence for improving patient outcomes [10, 23–25]. This lack of specificity may hinder the ability to effectively measure and interpret the impact of certain clinical activities on the quality of care, as the relevant components of care quality are not specified [34, 35]. Nevertheless, the lack of specific linkage may be because some clinical pharmacy activities overlap in practice, making it difficult to determine the contribution of individual tasks to specific outcomes. For example, a pharmacist may perform medication reviews and clinical reviews simultaneously to determine the appropriateness of prescribed medication [10]. Research investigating clinical pharmacy quality indicators suggests that meaningful care outcomes are better realized when multiple clinical activities are collectively measured, as single clinical interventions are unlikely to result in meaningful outcomes when performed alone in complex healthcare settings [43, 44]. Therefore, to inform clinical governance on the contribution of clinical pharmacy services to organizational care goals, it may be appropriate to measure the impact of collectively performing the recommended clinical pharmacy activities on relevant care outcomes.

It is important to acknowledge that some recommended measurements to evaluate pharmacy services are not exclusively influenced by pharmacy staff. Other health professionals participate in the pharmaceutical care process in hospitals and may also perform medication histories or reviews [45–47]. Thus, to support meaningful evaluation of the quality of pharmacy service utilizing the recommended measures, it would be important to focus measurements on clinical activities performed by pharmacy staff, how they are performed and the relevant outcomes [48, 49]. Without this distinction, assessments may conflate the quality of pharmacy service provision with the broader outcomes of pharmaceutical care, which may obscure the effect of pharmacy services [48, 50].

It may be feasible to perform regular measures of the recommended clinical pharmacy activities and outcomes using information that is automatically recorded in electronic systems. Electronically captured information generally requires fewer staffing resources to facilitate data collection, and databases yield large volumes of data that can be reproduced [51, 52]. Information reflecting clinical pharmacy activities, medication incidents and quality improvement activities may be recorded electronically in hospitals that utilize electronic medication management systems [10, 24]. Activities that are regularly performed by pharmacists, such as patient-specific clinical pharmacy activities, may demonstrate clinical accountability and provide evidence for the impact of pharmacy services if routinely documented in patient records [10, 12]. It may be most appropriate to explore the use of these data for governance by capturing patient-specific clinical pharmacy activities and their associated outcomes.

### Strengths and limitations

A major strength of this study is the use of widely endorsed policy documents from reputable organizations across international practice settings, which enhances the generalizability and relevance of the findings. By systematically examining these documents, the study identifies a defined set of practice-specific activities that can potentially guide routine monitoring and evaluation in hospital pharmacy services. This study also indicates how these activities may align with broader organizational objectives for healthcare delivery. In addition, this study contributes to understanding what defines “quality” when evaluating the provision of clinical pharmacy services in hospitals, and highlights the need for a balanced and comprehensive approach due to the potential over- or under-prioritization of certain components.

This study has limitations. Document selection and analysis were conducted by a single investigator, which may introduce subjectivity in categorizing desirable care elements and recommended clinical governance measures. However, this was minimized by employing a structured framework for document selection and a systematic approach for document analysis. This study only included documents that were published in English by organizations from high-income countries. As such, clinical governance approaches from countries of different income levels and other language groups may not be accounted for in this study.

## Conclusions

It is widely recommended for hospital pharmacy services to be patient-centred, safe, effective, integrated, timely, equitable, reliable and efficient, to deliver high-quality pharmaceutical care that maximizes positive outcomes for patients. Regularly measuring and monitoring the extent to which these desirable elements of care are evident in the delivery of clinical pharmacy services may help to understand how services are delivered and inform clinical governance on quality improvement initiatives. Future research should investigate the practicality and suitability of utilizing data from electronic systems in hospitals to continuously monitor the delivery, impact and outcomes of pharmacy services.

## Supplementary Information

Additional file 1 (Search terms entered in the websites of national or multinational healthcare and pharmacy organisations)

Additional file 2 (Lists of the desirable elements of clinical care delivery identified in the review of national clinical governance frameworks and pharmacy governance documents.)

Additional file 3 (List of clinical activities completed by hospital pharmacists)

## Data Availability

The authors confirm that the data supporting the findings of this study are available within the article and/or its supplementary materials.
