# Supplementary material for: Elements of healthcare delivery required to facilitate the clinical governance of hospital pharmacy services: a document review
Source: Health Res Policy Syst. 2025 Aug 4;23:100. doi: 10.1186/s12961-025-01378-w (PMC12323264; doi:10.1186/s12961-025-01378-w)
Supplement: Supplementary file 1 — Additional file 1 (Search terms entered in the websites of national or multinational healthcare and pharmacy organisations) [file 12961_2025_1378_MOESM1_ESM.docx]

# Additional File 1:

| **Supplementary Table 1:** Search terms entered in the websites of national or multinational healthcare and pharmacy organisations |
| --- |
| **Search terms used** |
| “Clinical governance”, “Governance framework”, “Clinical governance framework”, “Clinical governance strategy”, “Clinical governance guidance”, “Quality and safety framework”, “Quality and safety strategy”, “Quality and safety guidance”, “Clinical pharmacy governance”, “Pharmacy governance framework”, “Practice standards”, “Practice guidelines”, “Clinical pharmacy practice guidelines”, “Clinical pharmacy practice guidance”, “Clinical pharmacy governance strategy” |
