# Supplementary material for: Elements of healthcare delivery required to facilitate the clinical governance of hospital pharmacy services: a document review
Source: Health Res Policy Syst. 2025 Aug 4;23:100. doi: 10.1186/s12961-025-01378-w (PMC12323264; doi:10.1186/s12961-025-01378-w)
Supplement: Supplementary file 2 — Additional file 2 (Lists of the desirable elements of clinical care delivery identified in the review of national clinical governance frameworks and pharmacy governance documents.) [file 12961_2025_1378_MOESM2_ESM.docx]

| **Supplementary Table 2:** Desirable elements of healthcare delivery identified in national clinical governance frameworks | | | | | |
| --- | --- | --- | --- | --- | --- |
|  | *National Model of Clinical Governance Framework*  (ACSQHC) | *Clinical Governance – guidance for health and disability providers* (NZHQSC) | *A Shared Commitment to Quality for Those Working in Health and Care Systems* (NQB) | *The Canadian Quality & Patient Safety Framework for Health Services* (HSO & CPSI) | *A Framework for Safe, Reliable, and Effective Care* (IHI) |
| **Quality** | p9 | p7 | p3 | p2 | p4, p6, 18 |
| **Patient-centred** | p6 | p13 | p3 | p6 | p18, p8, p25 |
| **Safe** | p33, p2 | p17 | p3 | p5 | p6, p18 |
| **Effective** | p6, p22 | p15 | p3 | p5 | p6 |
| **Equitable** |  | p7 | p3 | p5 | p4 |
| **Efficient** |  | p7 | p3, p10 | p52 | p18, p19 |
| **Integrated** | p2 | p6, p19 | p4 | p9 | p14 |
| **Reliable** | p1 | p15, p17, p19 | p3 | p5 | p18 |
| **Timely** |  | p7 |  | p5 | p17 |

# Additional File 2:

| **Supplementary Table 3:**  Desirable elements of healthcare delivery identified in pharmacy documents | | | | | | | | | | | |
| --- | --- | --- | --- | --- | --- | --- | --- | --- | --- | --- | --- |
|  | *Standards of Practice for New Zealand Hospital*  *Clinical Pharmacy Services*  (NZHPA) | *National Pharmacist Services*  *Framework*  *2014*  (PSNZ) | *Standards of Practice for Clinical Pharmacy Services*  (SHPA) | *Pharmacy Practice in Hospitals and Other Collaborative Healthcare Settings*  (CSHP) | *Revised FIP Basel Statements on the Future of Hospital Pharmacy*  (FIP) | *European Statements of Hospital Pharmacy* (EAHP) | *ASHP Guidelines: Minimum Standards for Pharmacies in Hospitals* | *Standards of Practice for Clinical Pharmacists*  (ACCP) | *Professional Standards for Hospital*  *Pharmacy Services* (RPS) | *Clinical Governance Principles*  *for Pharmacy Services 2018*  (PSA) | *Hospital Pharmacists: Information Paper on Enhancing Quality and Safety in Medication Use* (CSHP) |
| **Quality** | p12 | p23 | s47 | p11 | p5 | p3 | p1 | p3 | p4 | p1 | p3 |
| **Patient-centred** | p34 | p4 | s47 | p4 | p1 | p1, p3 | p2 | p3 | p4 | p12, p14 | p3 |
| **Safe** | p1 | p5 | s3 | p5 | p1 | p1 | p1 | p2 | p4 | p10 | p3 |
| **Effective** | p34 | p3 | s47 | p5 | p1 | p1 | p1 | p2 | p4 | p10, p13 | p3 |
| **Equitable** | p12 | p4, p17 | s30 | p5 |  |  |  |  |  | p1 | p3 |
| **Efficient** |  |  |  |  |  | p1 | p2 | p3 | p19 | p1, p11 | p3 |
| **Integrated** | p34 | p3 | s3 | p6 | p1 | p1, p3 |  | p1, p3 | p11, p13 | p16 | p9 |
| **Reliable** | p12 |  |  | p11 |  |  | p1 |  |  | p16 | p18 |
| **Timely** |  | p10 | s4, s30 | p6 | p1 | p3 | p5 |  | p9, p10, p13 | p1 | p3 |
