# Supplementary material for: Elements of healthcare delivery required to facilitate the clinical governance of hospital pharmacy services: a document review
Source: Health Res Policy Syst. 2025 Aug 4;23:100. doi: 10.1186/s12961-025-01378-w (PMC12323264; doi:10.1186/s12961-025-01378-w)
Supplement: Supplementary file 3 — Additional file 3 (List of clinical activities completed by hospital pharmacists) [file 12961_2025_1378_MOESM3_ESM.docx]

# Additional File 3:

| Supplementary Table 4: Clinical activities completed by hospital pharmacists | | |
| --- | --- | --- |
| Classification | **Activity** | **Description** |
| Patient-specific Pharmacist Activities | Medication history | An accurate record of the medications taken by a patient before admission is obtained through consultations with patients, carers, community pharmacies, and other relevant sources. |
|  | Medication reconciliation | Medications prescribed in the hospital are compared to a patient’s medication history to identify and resolve discrepancies. |
|  | Medication review | Systematic evaluation of the medications prescribed for a patient in the hospital to assess the appropriateness and safety of treatment and to optimise the medication regimen according to the patient’s care goals. |
|  | Clinical review | Assessment of patient-specific clinical information such as biochemical parameters or other test results to evaluate their response to medications or identify and manage medication-related issues. |
|  | Adverse drug reaction management | Monitoring the use of medications to specifically identify, assess, manage, and prevent, noxious and unintended responses to medications administered at normal treatment doses. |
|  | Medication optimisation & management plans | In partnership with patients and other healthcare providers, pharmacists create and update a plan that outlines ongoing medication management for individual patients. |
|  | Medication prescribing | Accredited pharmacists work collaboratively in a treating team to prescribe medications within the limits of their professional and clinical competence. |
|  | Medication administration | Accredited pharmacists administer medications to patients in the hospital. |
|  | Therapeutic drug monitoring | Pharmacists monitor and interpret the concentration of a medication or its surrogate markers in a patient’s body fluids to ensure patient-specific optimisation of the safety and effectiveness of medication therapy. |
|  | Documentation of care delivery | Pharmacists document clinical activities in patients’ records to demonstrate accountability for their clinical duties and provide evidence for the outcomes of care. |
|  | Patient counselling and education | Pharmacists provide evidence-based information and advice on medication-related matters to patients to promote the safe and appropriate use of their medications. |
|  | Provision of discharge services | Includes all the activities that a pharmacist may perform when a patient is transitioning care to ensure the patient has the correct medications, appropriate medication information, ongoing medication access, and referral to relevant health professionals for ongoing review and monitoring of their medications. |
| Patient-specific Health Team Activities | Medical rounds | Pharmacists partake in clinical ward rounds led by the caring physician team to review and plan the care for individual patients. |
|  | Multidisciplinary meetings | Pharmacists participate in clinics or meetings, where other health professionals are present to facilitate multidisciplinary care planning for their patients. |
| Non-patient-specific Pharmacist Activities | Development of medication guidelines and protocols | Pharmacists participate in the development and maintenance of evidence-based medication guidelines, procedures, and protocol documents to guide practice. |
|  | Research | Pharmacists conduct research with a focus on clinical outcomes in relation to medications and aim to contribute to improving clinical pharmacy practice. |
|  | Drug use evaluations | Pharmacists compare the use of medications to best practice guidelines and benchmarks to understand how the systems are performing. |
|  | Antibiotic stewardship | Participate in activities that evaluate the use of antibiotics to promote safety, and appropriate use that aligns with best practice guidelines. |
|  | Medication information to other health Services | Provision of evidence-based information and advice on medication-related matters to health professionals or health services to promote safe and appropriate use of medications. |
|  | In-service training and education | Pharmacists provide education and training to other pharmacy staff or health professionals or participate in continuous professional development programs. |
